# Anti-inflammation and anti-apoptosis effects of growth arrest-specific protein 6 in acute liver injury induced by LPS/D-GalN in mice ^1^

**DOI:** 10.1590/s0102-865020200020000004

**Published:** 2020-04-09

**Authors:** Qian Wang, Yang Zhao, Bin Zang

**Affiliations:** I Master, Department of Emergency Medicine, 4th Affiliated Hospital of China Medical University. Student, Department of Critical Care Medicine, Shengjing Hospital, China Medical University, Shenyang, China. Technical procedures, interpretation of data, statistical analysis, manuscript preparation.; Department of Critical Care Medicine, Shengjing Hospital, China Medical University, Shenyang, China; II MD, Department of Critical Care Medicine, Shengjing Hospital of China Medical University, Shenyang, China. Acquisition and interpretation of data, statistical analysis, critical revision.; III Master, Chairman and Head, Department of Critical Care Medicine, Shengjing Hospital of China Medical University, Shenyang, China. Conception and design of the study, critical revision, supervised all phases of the study.

**Keywords:** Chemical and Drug Induced Liver Injury, Sepsis, Mice

## Abstract

**Purpose:**

To investigate the effect of growth arrest-specific protein 6 (Gas6) on acute liver injury in mice and related mechanisms.

**Methods:**

Thirty C57BL/6 (6-8 weeks old) mice were randomly divided into control, LPS/D-GalN, and LPS/D-GalN+Gas6 groups (10 mice in each group). The LPS/D-GalN group was intraperitoneally administered with LPS (0.25 mg/Kg) and D-GalN (400 mg/Kg) for 5h. The LPS/D-GalN+Gas6 group was intraperitoneally administered with rmGas6 one hour before intraperitoneal application of LPS/D-GalN. All subjects were sacrificed at 5 h for blood and tissue analysis. The expression of protein and mRNA was assessed by western blotting and RT-PCR, respectively.

**Results:**

Compared with the control group, AST, ALT, IL-1β, TNF-α, IL-6 IL-10, MPO activity were increased in the LPS/D-GalN group. However, they were significantly inhibited by Gas6. Gas6 markedly suppressed the expression of apoptosis-related protein induced by LPS/D-GalN. Moreover, Gas6 attenuated the activation of the NF-κB signaling pathway in acute liver injury induced by LPS/D-GalN.

**Conclusions:**

Gas6 alleviates acute liver injury in mice through regulating NF-κB signaling pathways. Gas6 can be a potential therapeutic agent in treating LPS/D-GalN-induced acute liver injury in the future.

## Introduction

Sepsis is a life-threatening condition caused by a dysfunctional response to infection and is the leading cause of death in the intensive care unit (ICU)^1^ . Sepsis is a significant public health problem due to its high morbidity and mortality rates, which are associated with hemodynamic damage, multiple organ dysfunction, and unconstrained inflammation^2 , 3^ . In the course of sepsis, the liver may be a potential target for dysregulation of the inflammatory response^4^ .

Although the clinical treatment of sepsis has progressed, its morbidity and mortality are still high^5^ . Hepatic dysfunction has specific prognostic relevance to the course of sepsis and is a powerful independent predictor of mortality. Improving liver function can reduce morbidity and mortality in patients with sepsis. It is a major challenge to discover effective and safe therapy of sepsis-induced acute liver injury. Currently, few studies have led to substantial advances in drug development or therapy. Therefore, there is an urgent need for a safe and effective treatment strategy for acute liver injury caused by sepsis.

Growth arrest-specific protein 6 (Gas6) and its tyrosine kinase TAM receptors (Tyro3, Axl, and Mer) are involved in growth and survival processes during tissue repair and development^6^ . In the liver, Gas6 is mainly expressed in Kupffer cells^7^ , and the expression of Gas6 is related to the severity of the disease in patients with septic shock, especially with kidney and liver dysfunction. Gas6 is a vitamin K-dependent protein with many functions. Although Gas6 and protein S have common characteristics, their biological effects are significantly different. Gas6 rarely participates in the coagulation cascade. It is mainly involved in cell protection and tissue formation and has extensive inhibition of inflammation.

Numerous studies have shown that gas6 can improve antioxidant capacity and inhibit inflammatory responses. Most previous studies have focused on ischemia-reperfusion models^8^ . Few studies have systematically reviewed the protection of gas6 against LPS/D-GalN-induced acute liver injury. We designed the study of Gas6 on acute liver injury to reveal its hepatoprotective effect and underlying mechanisms.

## Methods

### Chemicals

LPS, D-galactosamine were purchased from Sigma-Aldrich Co (St. Louis, MO, USA). AST, ALT, MPO kits were purchased from the Institute of Jiancheng Bioengineering (Nanjing, China). TNF-α, IL-1β, and IL-6 ELISA kits were purchased from Biolegend (San Diego, CA, USA). Gas6 was purchased from R&D Systems (Minneapolis, MN, USA). Antibodies used for western blot were as follows: anti-Bcl-2 (Proteintech, Rosemont, IL, USA), anti-Bax (Proteintech, Rosemont, IL, USA), anti-ß-actin (Proteintech, Rosemont, IL, USA), NF-κB signal pathway kit (Cell Signaling Technology, Beverly, MA, USA).

### Animals and treatment

6-8 weeks male C57BL/6 mice were purchased from the Experimental Animal Center of China Medical University. Mice were housed in a specific-pathogen-free facility under a 12 h light-dark cycle. All animals’ procedures were performed according to protocols approved by the Institutional Animal Care and Use Committee of China Medical University (Grant No. 2019PS563K).

C57BL/6 mice were randomly divided into control, LPS/D-GalN, and LPS/D-GalN+Gas6 group, 10 mice in each group. The LPS/D-GalN group was intraperitoneally administered with LPS (0.25 mg/Kg) and D-GaIN (400 mg/Kg). LPS/D-GalN+Gas6 group was intraperitoneally administered with rmGas6 (250 ug/Kg) one hour before intraperitoneal administration of LPS (0.25 mg/Kg) and D-GaIN (400 mg/Kg). After 5 hours of LPS/D-GalN injection, mice were sacrificed under anesthesia for experimental evaluation. Blood was collected from the abdominal aorta, and the liver was dissected. The protein and total RNA were extracted immediately from the liver and frozen at -80°C.

### ALT/AST activities and inflammatory cytokines

Blood samples were collected and centrifugated at 5000 rpm for 10 min. Serum aspartate aminotransferase (AST and ALT) activities were analyzed by using the commercial kits (Jiancheng Bioengineering Institute, Nanjing, China) following the instructions by the manufacturer.

Blood samples were collected for the detection of TNF-α, IL-1β, IL-10, and IL-6 levels using commercial kits produced by Biolegend (San Diego, CA, USA), following the instructions of the manufacturer. The concentrations of inflammatory cytokines were calculated by generating a standard curve.

### MPO assay

Neutrophils are the core cells of oxidative stress, which release reactive oxygen species (ROS) and increase oxidative stress. Tissue myeloperoxidase activity can be used as an indicator of neutrophil infiltration. 5 h after administration of LPS/D-GalN, liver tissue was homogenized in PBS solution (20 mmol/L, pH 7.4) and centrifuged at 30,000 g for 30 minutes. The precipitate was redissolved in potassium phosphate buffer (50 mmol/L, pH 6.0) with 0.5% hexadecyltrimethylammonium bromide. The supernatant was taken after centrifugation at 20,000 g for 15 min. MPO activity in the liver was determined by absorbance change at 460 nm with spectrophotometric.

### Western blotting

The liver tissue was taken to extract total and nuclear proteins by commercially kits (Beyotime, China) according to the manufacturer’s instructions and denatured with 5× loading buffer at 95°C for 5 min. After the protein concentration was determined by BCA protein estimation kit (Beyotime, China), the sample was diluted to the same concentration. Equal quantities of protein samples were fractionated by SDS-PAGE and then transferred to the PVDF membrane. The membranes were overnight incubated with primary antibodies against Bcl-2 (1: 1000), Bax (1: 1000), IκBα (1: 1000), NF-κB (1: 1000), p-NF-κB (1: 1000), β-actin (1:1000). The secondary antibody (1: 2000) was incubated for 2 h at room temperature. A chemiluminescent peroxidase substrate (ECL; Beyotime, China) was applied according to the manufacturer’s instructions.

### Quantitative real-time PCR

Total RNA from the mouse’s liver was isolated with RNAiso Plus (TaKaRa, Dalian, China) and reverse-transcribed into complementary DNA by using a PrimeScriptTM RT reagent Kit (TaKaRa, Dalian, China). SYBR Premix Ex Taq (TaKaRa, Dalian, China) was used to perform the PCR reaction following the instructions of the manufacturer. The primer sequences were as follows: Bax Forward: ATGCGTCCACCAAGAAGC, Reverse: CAGTTGAAGTTGCCATCAGC. Bcl-2 Forward: AGCCTGAGAGCAACCCAAT, Reverse: AGCGACGAGAGAAGTCATCC.β-actin Forward: CGTGAAAAGATGACCCAGATCA, Reverse: TGGTACGACCAGAGGCATACAG. Quantitative real-time PCR was performed using the Applied Biosystems 7500 Real-Time System. The comparative CT method (2−ΔΔCt) was used to determine the relative quantification of target genes normalized to that of β-actin.

### Statistical analysis

The data are expressed as the means ± SE at least three independent experiments and compared by the one-way analysis of variance (ANOVA) and the Student Newman-Keuls test using the Prism 7 software. P-values less than 0.05 were considered as statistically significant.

## Results

### Gas6 attenuates AST and ALT activity induced by LPS/D-GalN

**Figure 1:**
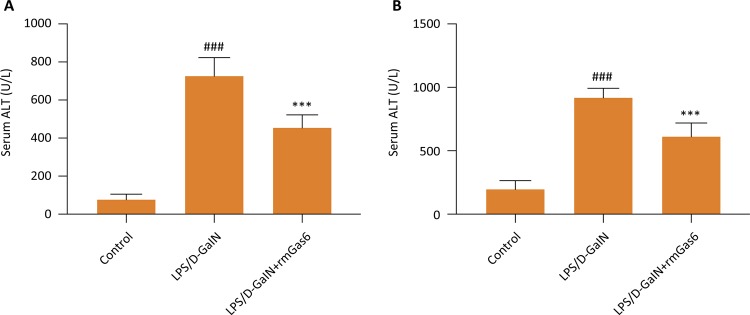
Gas6 repressed the activities of ALT and AST in acute liver injury induced by LPS/D-GalN. ALT and AST activities were evaluated by using commercial assay kit according to the manufacturer’s protocols. *** *p* < 0.01 compared to LPS/D-GalN group. ### *p* < 0.01 compared to control group.

Serum AST and ALT were assessed to observe the effect of Gas6 on LPS/D-GalN-induced liver injury. As shown in Figure 1 , the ALT and AST activities of the LPS/D-GalN group were increased dramatically. However, Gas6 significantly inhibits AST and ALT activity in liver injury induced by LPS/D-GalN ( *p* <0.01).

### Gas6 significantly reduces the production of inflammatory cytokine induced by LPS/D-GalN

**Figure 2:**
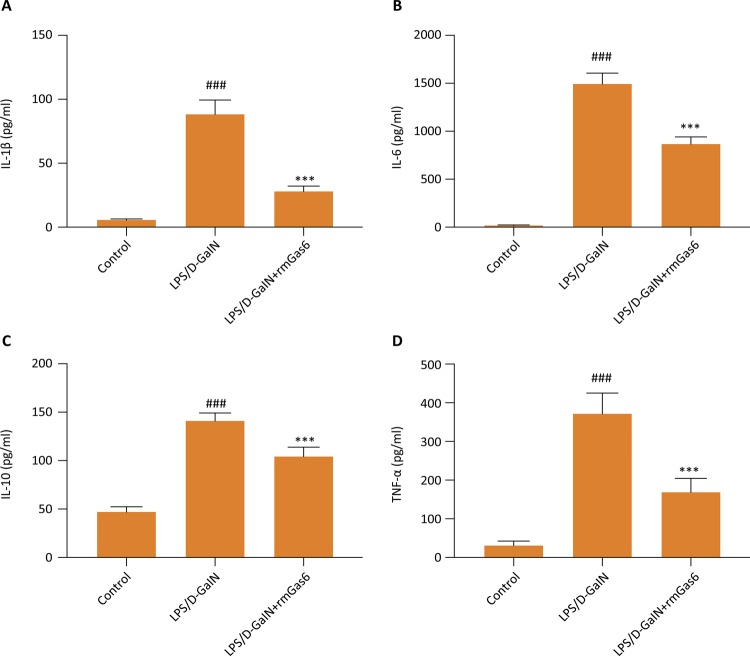
Effect of Gas6 on IL-1ß (A), IL-6 (B), IL-10 (C), and TNF-α (D). Quantitation of IL-1ß, IL-6, IL-10, and TNF-α were performed by ELISA. Data are represented as mean ± SE (n=10). *** *p* < 0.01 compared to LPS/D-GalN group. ### *p* < 0.01 compared to control group.

The prominent manifestation of sepsis is an excessive inflammatory response to infection. We measured serum inflammatory cytokine, including TNF-α, IL-1β, IL-10, and IL-6 at 5 hours after administration of LPS/D-GalN. The LPS/D-GalN group had significantly increased levels of TNF-α, IL-1β, IL-10, and IL-6, whereas Gas6 inhibited the overproduction of these inflammatory cytokines ( Fig. 2 , *p* < 0.01).

### Gas6 inhibits activation of MPO induced by LPS/D-GalN

**Figure 3:**
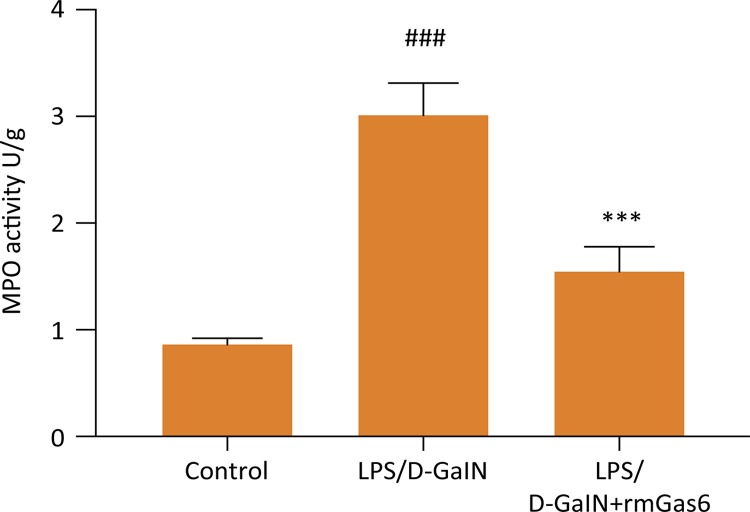
Effect of Gas6 on LPS/D-GalN-induced liver injury. Leukocyte infiltration was evaluated by using MPO assay kit according to the manufacturer’s protocols. *** *p* < 0.01 compared to LPS/D-GalN group. ### *p* < 0.01 compared to control group.

Tissue myeloperoxidase activity can be used as an indicator of neutrophil infiltration, which releases reactive oxygen species (ROS) and causes oxidative stress. As shown in Figure 3 , the MPO activities of the LPS/D-GalN group were increased dramatically. However, Gas6 significantly inhibits MPO activity in liver injury induced by LPS/D-GalN ( *p* <0.01).

### Gas6 inhibits the expression of proteins induced by LPS/D-GalN

**Figure 4:**
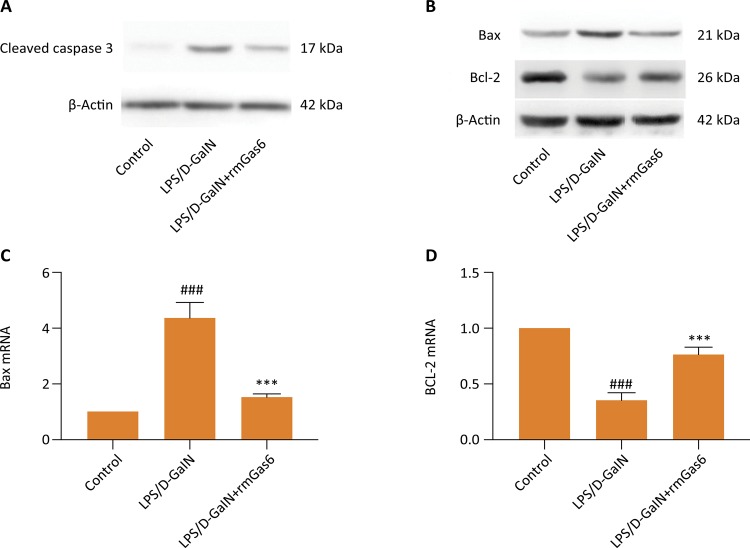
Gas6 inhibited the apoptosis of the liver. Western blot and RT-PCR to determine the effect of Gas6 on apoptosis-related proteins. (A, B) Western blot of cleaved caspase 3, Bax and Bcl-2 in different groups. (C, D) RT-PCR of Bcl-2 and Bax in different groups. *** *p* < 0.01 compared to LPS/D-GalN group. ### *p* < 0.01 compared to control group.

To explore the effect of Gas6 on LPS/D-GalN-induced hepatocyte apoptosis, relevant proteins (Caspase-3, Bax, and Bcl-2) were examined by western blot. As shown in Figure 4 , Bax increased, and Bcl-2 expression decreased in the LPS/D-GalN group. Downregulation of Bax and upregulation of Bcl-2 were found in the LPS/D-GalN +Gas6 group ( Fig. 4 B-D ). Furthermore, caspase-3 was elevated at 5 h after administration of LPS/D-GalN. However, the increase of caspase-3 was also repressed in Gas6 pretreated group ( Fig. 4A ).

### Gas6 inhibits activation of the NF-κB signaling pathway induced by LPS/D-GalN

**Figure 5:**
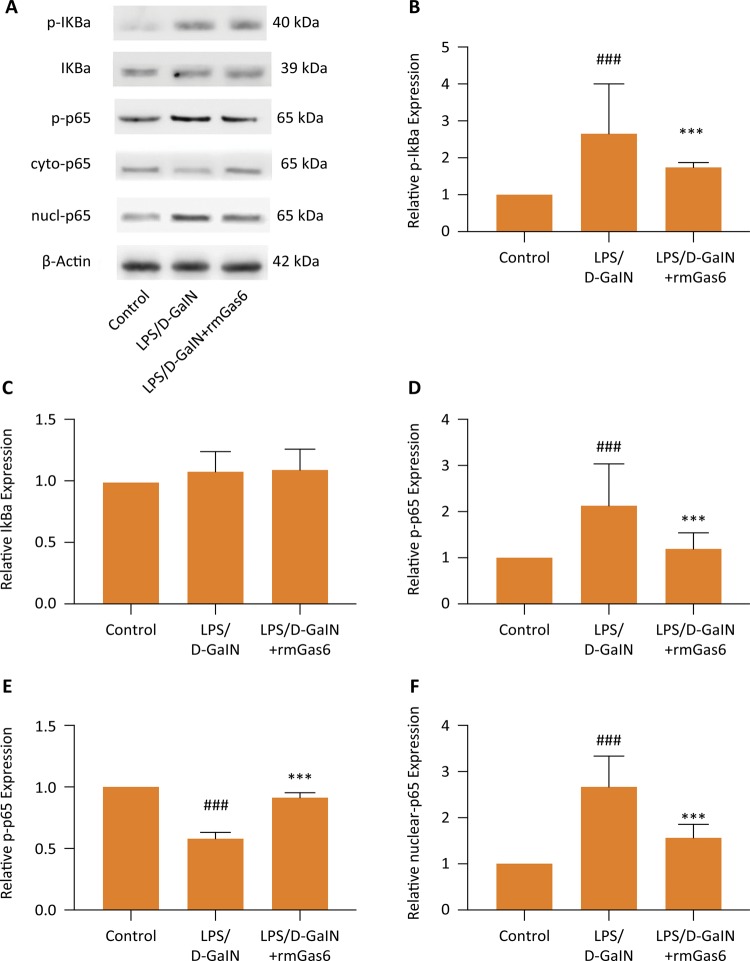
Gas6 inhibited the activation of NF-κB induced by LPS/D-GalN. The expression of related proteins of the NF-κB signaling pathway were detected by western blot. The results shown are representative of at least three independent experiments. *** *p* < 0.01 compared to LPS/D-GalN group. ### *p* < 0.01 compared to control group.

Western blot analysis of critical molecules in the NF-κB pathway. As shown in Figure 5 , in the LPS / D-GalN group, expression of p-IκBα, p-p65, and NF-κB in the nucleus were significantly increased in the CLP and CLP+PBS groups. However, Gas6 can reduce the activation of the NF-κB signal transduction pathway.

## Discussion

Gas6 is a vitamin K-dependent secreted protein consisting of 678 amino acids with a relative molecular mass of 75 kDa and has 43% of the same sequence with protein S^9^ . It is a common ligand for Tyro3, Axl, and Mer^10 - 12^ . Gas6 and its receptors are expressed in macrophages, granulocytes, dendritic cells, and endothelial cells and vascular smooth muscle. Gas6 works by stimulating phagocytosis, reducing the inflammatory response, limiting hypoxic cell damage, and enhancing cell proliferation^13 - 21^ . Gas6 activation of the TAM receptor produces meaningful therapeutic targets in thromboembolic diseases, atherosclerosis, sepsis, autoimmune diseases, and cancer^22 , 23^ .

Sepsis is a dysregulated host response caused by an infection and often affects multiple organs, such as the liver. The impact in the clinical setting is significant^24^ . There is an urgent need to study and use novel mechanisms to avoid organ damage from sepsis^25^ . LPS/D-GalN induced acute liver injury is a widely accepted experimental model that closely resembles acute liver injury seen clinically^26^ . In this in vivo model, LPS/D-GalN activates a complex inflammatory response. ALT and AST are abundant in liver cells. They are a sensitive and specific marker of liver injury^27^ . Intraperitoneal administered with LPS (0.25 mg/Kg) and D-GalN (400 mg/Kg) for 5h markedly increased serum levels of ALT and AST confirming that acute liver injury occurred. By using a mouse model treated with LPS/D-GalN, we demonstrated that pretreatment with Gas6 could protect against liver injury. Gas6 pretreatment can reduce clinical parameters of liver function (AST and ALT), apoptosis-related proteins, inflammatory cytokines.

Inflammatory cell infiltration has shown to be involved in LPS / D-GalN-induced acute liver injury. In our study, MPO activity was used to measure neutrophil infiltration, and we found that Gas6 treatment reduced inflammatory infiltration compared with that in LPS/D-GalN group. Similarly, the level of a direct indicator of the inflammatory response was also significantly reduced by the administration of Gas6. In our study, Gas6 treatment also showed a noticeable effect of preventing the over-production of IL-1, IL-6, TNF-a, IL-10. In the meanwhile, we also noted a significant decrease of apoptotic protein in the liver after LPS/D-GalN with Gas6 treatment. The important role of Gas6 is to enhance macrophages to clear apoptotic cells. Failure to effectively clear apoptotic cells will develop secondary necrosis, which will lead to the occurrence of the inflammatory response and further tissue damage. Therefore, reducing liver cell apoptosis may be one of the mechanisms for exogenous supplementation of Gas6 to reduce LPS / D-GalN-induced liver injury.

We selected the NF-κB signaling pathway as the potential anti-inflammatory mechanism of Gas6 on LPS / D-GalN-induced acute liver injury. NF-κB activation is the key to the excessive inflammatory response that causes sepsis-related organ dysfunction^28 , 29^ . P65 is an important subunit of NF-κB. The activation level of p65 is often used to indicate the degree of activation of NF-κB^30^ . LPS triggers the activation of NF-κB translocated into the nucleus, resulting in the expression of proinflammatory cytokines, such as IL-1β, IL-6, TNF-α. The released cytokines can also activate NF-κB in turn, forming a positive feedback loop. In the present study, the NF-κB signal pathway was activated by LPS/D-GalN. The phosphorylation of p-65 was downregulated after Gas6 treatment. NF-κB regulates the expression of multiple genes in the early inflammatory response, and these genes are involved in the inflammatory response and the occurrence of acute liver injury. Our study found that Gas6 inhibits the activation of the NF-κB signaling pathway caused by LPS / D-GalN. Based on the above findings, the protective effect of Gas6 on LPS / D-GalN-induced acute liver injury in mice may be related to inhibition of the NF-κB signaling pathway.

## Conclusions

Gas6 can alleviate liver injury in mice. A possible mechanism is that Gas6 inhibits the activation of the NF-κB signaling pathway. This study provides a basis for elucidating the protective mechanism of Gas6 against acute liver injury.
